# Two new species of *Byrrhinus* Motschulsky, 1858 (Coleoptera, Limnichidae, Limnichinae) from Negros, Philippines

**DOI:** 10.3897/zookeys.1070.70531

**Published:** 2021-11-10

**Authors:** Emmanuel D. Delocado, Hendrik Freitag

**Affiliations:** 1 Ateneo Biodiversity Research Laboratory, Department of Biology, School of Science and Engineering, Ateneo de Manila University, Quezon City, 1108 Philippines Ateneo de Manila University Quezon City Philippines

**Keywords:** Biodiversity assessment, COI, integrative taxonomy, minute marsh-loving beetle, Negros Island, new species

## Abstract

Two new species of Limnichidae beetles, *Byrrhinusnegrosensis***sp. nov.** and *Byrrhinusvillarini***sp. nov.**, are described from the Island of Negros in the Philippines. The adult specimens of the new species can be differentiated by patterns of body punctation, colour and orientation of elytral pubescence, posterolateral angle of pronotum, tarsomere length ratio and aedeagal form. Two clades, representing the two new species, were retrieved in the Maximum Likelihood gene tree using the 3’-end of the *COI* gene. Maximum genetic divergence within *B.negrosensis* sp. nov. and *B.villarini* sp. nov. were recorded to be 2.3% and 1.3%, respectively, while the mean interspecific divergence between the two new species was 19.7%. Morphological descriptions, digital photographs and *COI* sequences were provided for the two species. The state of knowledge of *Byrrhinus* is reviewed and an updated Philippine checklist is provided. By coupling morphological and molecular data, this paper provides the first additional new species of Philippine *Byrrhinus* in the last 28 years.

## Introduction

*Byrrhinus* Motschulsky, 1858 is the most speciose limnichid genus with currently at least 87 species (Yoshitomi, unpublished data). Approximately 20% of known limnichid species belong to the genus *Byrrhinus*. The distribution of the genus is pantropical, but it is lacking or not yet recorded in some regions (Hernando and Ribera 2016). Currently, *Byrrhinus* is one of the two genera recorded in both the Old and New Worlds, although notably absent in the Nearctic and Palearctic (Wooldridge 1987).

The distinguishing features of *Byrrhinus* include its elongate oval habitus and deeply bisinuate pronotum and elytral base (Wooldridge 1987), as well as having a spiculum which can be dismantled from the male aedeagus (Hernando and Ribera 2014b). Additionally, *Byrrhinus* species generally possess a long yellowish pubescence and a densely punctured pronotum and metasternum. Like most members of Limnichidae, larval and pupal stages of *Byrrhinus* are undescribed. The internal anatomy was documented by Hinton (1939).

The genus was originally erected in 1858 to contain *Byrrhinuslatus* Motschulsky, 1858 from India. Later on, Blackburn (1896), Sharp (1902) and Pic (1922, 1923) described species from Australia, Africa and America and classified them in different genera, including *Eulimnichus* Casey, 1889, “*Notiocyphon* Blackburn, 1896”, “*Cyphonichus* Sharp, 1902”, “*Byrrhininus* Pic, 1922” and “*Pelocherops* Pic, 1923” with the last four being synonymised with *Byrrhinus*. Some of the species originally described as “*Cyphonichus*”, however, belong instead to genus *Paralimnichus* Deléve, 1973. Valuable taxonomic revisions on the genus were undertaken by Champion (1923), Deléve (1973) and Wooldridge (1987) who provided re-descriptions and a key to New World species. Subgenera and species groupings were erected by Satô (1965) and Deléve (1968), respectively, though these were not widely used in more recent publications. The most recent contribution to the genus was a new species from Angola (Matsumoto 2021).

Currently, five *Byrrhinus* species have been recorded in the Philippines (Deléve 1973; Wooldridge 1993; Freitag et al. 2016), namely, *B.convexus* (Blackburn, 1896), *B.ferax* Wooldridge, 1993, *B.punctatus* (Pic, 1922), *B.subtestaceus* Pic, 1923 and *B.tarawakanus* Deléve, 1973. A key to Philippine species for *Byrrhinus* was provided by Wooldridge (1993). Of the five species, two are not endemic to the country, namely *B.convexus* which was recorded also from Australia and *B.ferax* which was also recorded from Malaysia. Currently, the known distribution of *Byrrhinus* in the Philippines is confined to the Islands of Luzon, Mindoro, Mindanao, Palawan and Tawi-Tawi with many islands being under-surveyed. In fact, Freitag et al. (2016) note that the currently known diversity of aquatic and riparian Coleoptera in the Philippines is less than half of the totally expected number of species which is partly due to very uneven sampling efforts throughout the Archipelago.

Species identification in limnichid beetles is challenging due to their cryptic nature and subtle interspecific phenetic differences. Congeneric species are typically differentiated through details in genitalia, pubescence and punctation. Thus, to accelerate species discovery of the almost cryptic *Byrrhinus* fauna in a megadiverse locality, this study uses DNA sequences to complement morphological descriptions. This approach, referred to as integrative taxonomy (Dayrat 2005; Will et al. 2005), has been performed in several aquatic and riparian insect groups to fast-track biodiversity documentation (Tänzler et al. 2014; Riedel and Tänzler 2016; Garces et al. 2020; Kodada et al. 2020; Sabordo et al. 2020).

This contribution presents two new species, *Byrrhinusnegrosensis* sp. nov. and *Byrrhinusvillarini* sp. nov., as supported by morphological and genetic data. Both species were collected from the Island of Negros in the central Philippine island group of Visayas. Recent extensive and collaborative sampling by the Ateneo Biodiversity Research Laboratory on this Island has led to the discovery of new aquatic insect species (Garces et al. 2020; Kaltenbach et al. 2020a, 2020b; Komarek and Freitag 2020; Sabordo et al. 2020).

## Materials and methods

### Taxon sampling

The organismic material used in this study was primarily retrieved in the scope of the School of Science and Engineering Industry 4.0 Research Fund (SI4-013) project on the freshwater macroinvertebrate diversity inventory by the Ateneo Biodiversity Research Laboratory of the Ateneo de Manila University, Quezon City, Philippines (AdMU). A light trap was set on riverbanks between 6:00 pm to 8:00 pm. Insects attracted to the black light were manually collected and stored in vials with 95% ethanol. Specimens were stored in a freezer (–20 °C) prior to their examination. Collections of the Ateneo Biodiversity Research Laboratory from previous field works of the second author were also examined and used for the molecular analysis of this study.

### DNA extraction and sequencing

Isolation of DNA was performed using the Qiagen DNeasy kit (Qiagen, Hilden, Germany) following the instructions of the manufacturer for animal tissues (Qiagen 2006). For all successful extractions, amplification of the 3’-end of cytochrome *c* oxidase subunit I (*COI-3*’) gene by polymerase chain reaction (PCR) was done using the primer pair Jerry (5’-CAACATTTATTTTGATTTTTTGG-3’) and Pat (5’-TCCAATGCACTAATCTGCCATATTA-3’) (Simon et al. 1994). The PCR temperature progression was set as 180 s at 94 °C; 35 cycles of 30 s at 94 °C, 60 s at 50 °C, 90 s at 72 °C; 300 s at 72 °C. Cleaning and sequencing of successful PCR products was done by Macrogen Europe.

### Phylogenetic analysis

The sequences of both complementary strands were traced manually using CHROMAS (Goodstadt and Ponting 2001) and their consensus sequences were generated using the software BioEdit v.7.2.5 (Hall 1999). Their ends were trimmed to generate a complete matrix of all sequences used. All publicly available *COI-3*’ sequences (Kundrata et al. 2017) for *Byrrhinus*, namely from Indonesia, Malaysia and Cameroon, as well as for *Limnichus* spp., were downloaded from NCBI BLAST (Altschul et al. 1990). *Limnichus*, also a member of subfamily Limnichinae, was chosen to serve as outgroup of the phylogenetic analysis (Table 1). All new and reference sequences were aligned using MUSCLE 3.7 (Edgar 2004). Sequences generated from this study were labelled as EDD### and were submitted to the Barcode of Life Database (BOLD; https://www.doi.org/10.5883/DS-BYRRHNEG). Table 1 provides the BOLD and NCBI GenBank accession numbers.

**Table 1.** GenBank and BOLD accession numbers of *COI-3*’ mtDNA sequences of specimens used in this analysis.

Species	Specimen code	Locality	Sex	GenBank	BOLD	GenSeq nomenclature
*Byrrhinusnegrosensis* sp. nov.	EDD116	Negros	female	OK316812	COLPH052-21	genseq-2 COI
	EDD119	Negros	male	OK316811	COLPH053-21	genseq-2 COI
	EDD122	Negros	male	OK316808	COLPH054-21	genseq-1 COI
	EDD123	Negros	male	OK316803	COLPH055-21	genseq-2 COI
	EDD127	Negros	male	OK316809	COLPH056-21	genseq-2 COI
	EDD270	Negros	male	OK316807	COLPH057-21	genseq-2 COI
*Byrrhinusvillarini* sp. nov.	EDD113	Negros	female	OK316817	COLPH058-21	genseq-2 COI
	EDD114	Negros	male	OK316815	COLPH059-21	genseq-2 COI
	EDD115	Negros	male	OK316804	COLPH060-21	genseq-2 COI
	EDD121	Negros	male	OK316813	COLPH061-21	genseq-1 COI
	EDD124	Negros	male	OK316818	COLPH062-21	genseq-2 COI
	EDD126	Negros	male	OK316805	COLPH063-21	genseq-2 COI
*Byrrhinusferax* Wooldridge, 1993	EDD067	Mindoro	male	OK316810	COLPH064-21	genseq-4 COI
*Byrrhinus* sp. A	EDD057	Palawan	male	OK316816	COLPH065-21	genseq-4 COI
*Byrrhinus* sp. B	EDD105	Luzon	male	OK316806	COLPH066-21	genseq-4 COI
*Byrrhinus* sp. C	EDD112	Mindanao	female	OK316814	COLPH067-21	genseq-4 COI
*Byrrhinus* sp.	UPOL RK0663	Cameroon		KX092882	GBCL40978-19	
*Byrrhinus* sp.	UPOL RK0664	Indonesia		KX092889	GBCL40979-19	
*Byrrhinus* sp.	UPOL RK0727	Malaysia		KX092888	GBCL40980-19	
*Limnichus* sp. (outgroup)	UPOL RK0666	Indonesia		KX092883	GBCL40986-19	
*Limnichus* sp. (outgroup)	UPOL RK0725	Malaysia		KX092886	GBCL40987-19	

Generation of a Maximum Likelihood (ML) tree with bootstrap analysis for 1000 replicates, as well as genetic distance computation using Kimura-2-paramter (K2P) model (Kimura 1980), was performed in MEGA 7 (Kumar et al. 2016). The ML tree was constructed using the best-fitted substitution model which was GTR+G+I (lnL = -3885.57). Visualisation of relationships amongst less divergent sequences for new species was done by constructing a TCS haplotype network (Clement et al. 2000) through statistical parsimony analysis as implemented in PopArt (Leigh and Bryant 2015).

### Morphological analysis

External morphology was examined by using an OLYMPUS SZ61 stereomicroscope (Olympus, Tokyo, Japan). The terminal abdominal portion of the specimens was dissected and treated overnight with lactic acid. The male aedeagi were then observed under an OLYMPUS CX21 compound microscope (Olympus, Tokyo, Japan). The specimen and its genitalia were glued on to entomologic paper for vouchering purposes. Photographs of vouchers were taken using a Canon EOS 6D with a macro lens and were stacked in Helicon Focus Pro v.7.6.1 (Helicon Soft, Kharkiv, Ukraine) and further processed using Adobe Photoshop CS6 (Adobe, San Jose, CA, USA). The aedeagi were digitally drawn over microscopic photograph underlays using Inkscape (GNU GPL, Boston, MA, USA).

Congeneric vouchers and type material at the Natural History Museum, Vienna, Austria (**NMW**) and Institute of Evolutionary Biology, Barcelona, Spain (**IBE**) were also examined.

Terminologies of the species’ descriptions follow the Limnichidae chapter of the Handbook of Zoology/Coloeptera (Hernando and Ribera 2016). Type specimens of the new species were deposited at the Philippine National Museum of Natural History, Manila, Philippines (**PNM**), Ateneo de Manila University, Quezon City, Philippines (**AdMU**) and Museum für Naturkunde Berlin, Germany (**ZMB**). Holotype labels were quoted verbatim from specimen labels with backslashes (\) indicating line break.

The following abbreviations were used:

**a.s.l.** above sea level;

**BS** bootstrap value;

**EL** elytra length;

**EW** elytra width;

**PL** pronotum length;

**PW** pronotum width;

**TL** total length.

## Results

### DNA sequence analysis

The alignment of *COI-3*’ sequences is composed of 723 bases with 221 parsimony-informative sites and 281 variable sites. No sequence in the matrix contains gaps, insertion, deletions or ambiguous sites. Sequences of *Byrrhinus* have low G-C concentration (16.5–20.2% C, 15.6–16.2% G).

Two clades were retrieved from the *Byrrhinus* specimens from Negros (Fig. 1), with each reciprocally monophyletic clade having representative specimens from two rivers about 200 km apart. The first clade, described here as *Byrrhinusnegrosensis* sp. nov., is strongly supported (BS = 100, Fig. 1) with an intraspecific distance ranging from 0 to 2.3% with an average of 1.3% (Table 2; Suppl. material 1: Table S1). Meanwhile, the second clade, described here as *Byrrhinusvillarini* sp. nov., is also strongly supported (BS = 100), but the maximum intraspecific distance (1.3%) is smaller than that in *B.negrosensis* sp. nov. The clade formed by *B.villarini* sp. nov. and *Byrrhinus* sp. from Malaysia appears to be strongly supported (BS = 100), but the genetic distance between the two populations averages at 5.9% (Table 2). The interspecific distance between the two Negros species ranges from 19.0 to 20.7% (mean = 19.7; Suppl. material 1: Table S1). With the exception of *B.villarini* sp. nov. and the sequence from Malaysia, the interspecific distance of the two new species from Negros with other Philippine *Byrrhinus* (12.8–20.0%) and publicly available sequences of non-Philippine *Byrrhinus* (19.5–29.0%) ranges from moderately high to high.

**Table 2.** Mean genetic distance of *Byrrhinus* specimens, based on partial *COI-3*’ sequences (in %).

#	Identity	*n*	1	2	3	4	5	6	7	8	9
1	*Byrrhinusnegrosensis* sp. nov.	6	1.3								
2	*Byrrhinusvillarini* sp. nov.	6	19.7	0.4							
3	*Byrrhinusferax* Wooldridge, 1993	1	13.2	13.8	–						
4	*Byrrhinus* sp. A	1	19.8	16.2	19.5	–					
5	*Byrrhinus* sp. B	1	16.6	15.6	19.4	17.7	–				
6	*Byrrhinus* sp. C	1	18.2	17.5	21.4	10.6	19.6	–			
7	*Byrrhinus* sp. (Malaysia)	1	20.0	5.8	17.0	16.4	17.0	13.7	–		
8	*Byrrhinus* sp. (Indonesia)	1	21.6	20.0	21.7	22.2	22.4	17.9	20.2	–	
9	*Byrrhinus* sp. (Cameroon)	1	25.16	28.2	28.6	24.7	25.0	26.5	28.9	23.4	–

**Figure 1.**
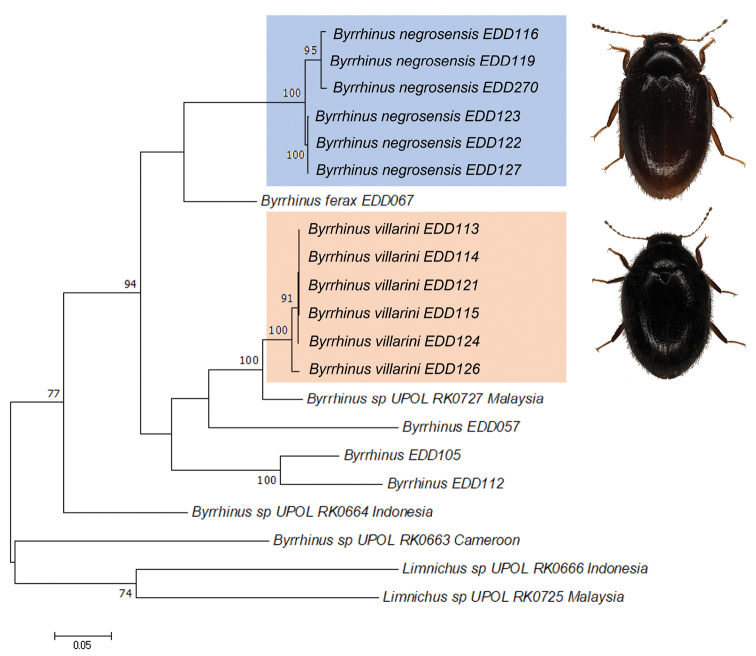
Maximum Likelihood gene tree using partial *COI-3*’, based on GTR+G+I parameter, 1000 bootstraps. Only bootstrap support values > 70% are indicated.

### Taxonomy

#### Family Limnichidae Erichson, 1847

**Subfamily Limnichinae Erichson, 1847**

##### Genus *Byrrhinus* Motschulsky, 1858

###### Byrrhinus negrosensis sp. nov.

Taxon classificationAnimaliaColeopteraLimnichidae

36510393-5CAB-5A80-A335-888518F27229

http://zoobank.org/95FDB6E9-6691-44BA-895A-D42954CA7086

Figures 2 , 4 , 5

####### Type locality.

Philippines • Negros Island, Negros Oriental, Valencia, Casaroro River, in secondary vegetation; ca. 09°18'N, 123°14'E; ca.150 m a.s.l.

####### Type material.

***Holotype*:** Philippines • ♂ (PNM: EDD122), “PHIL: Negros Or., Valencia, \ Casaroro River, downstr., sec.veg.; \ ca. 09°18'N; 123°14'E; ca.150 m a.s.l.; \ 01 Sep. 2019, leg. Garces & Pelingen (655)L”; GenBank: OK316808; BOLD: COLPH054-21; EDD122, habitus and terminal parts of abdomen including genitalia glued separately on to entomological card. ***Paratypes***: Philippines • 3♂♂ (ADMU: EDD123, EDD127): same data as holotype; GenBank: OK316803, OK316809; BOLD: COLPH055-21, COLPH056-21 • 3♂♂, 3♀♀: (ADMU: EDD116, EDD119, EDD270; PNM; ZMB) “PHIL: Negros Occ., Murcia, \ Pandanon River, sec.veg.; \ ca. 10°34'54"N; 123°10'30"E; ca. 440 m a.s.l.; \ 01 May 2019, leg. Freitag et al. (650)L”; GenBank: OK316807, OK316811, OK316812; BOLD: COLPH052-21, COLPH053-21, COLPH057-21.

####### Description.

**Figures 2, 3.**
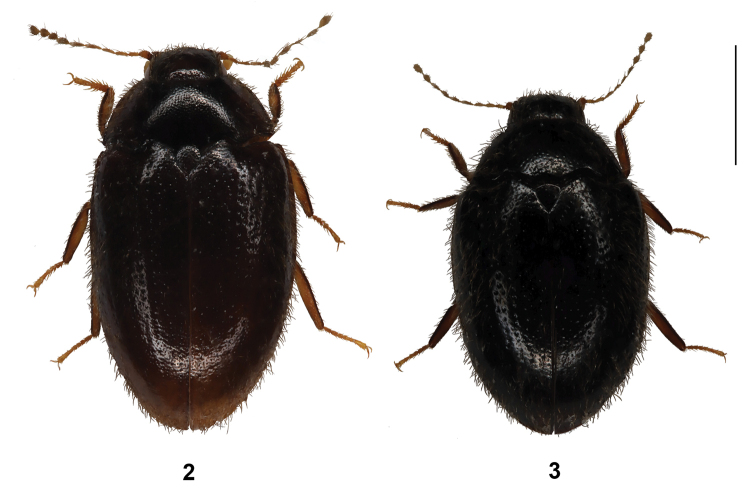
Habitus of new *Byrrhinus* species **2***Byrrhinusnegrosensis* sp. nov. **3***Byrrhinusvillarini* sp. nov. Scale bar: 1 mm.

***Body*:** (Fig. 2) elongate-oval, TL = 3.2 mm (2.9–4.1 mm), EW = 2.1 mm (1.8–2.3 mm), widest behind mid-leg; dorsal surface brown to dark brown; body appendages slightly paler than body, moderately densely and evenly covered with yellow–brown to brown, fine, quite long, mostly erect pubescence; antennae yellow-brown to brown; femora and tibiae brown; tarsi brown.

***Head*:** obscurely rugulose; broadly laminate over eyes; margins of frons grooved over eyes; sides of frons with deep and well–marked pit-like depressions. Punctation minute, sparse, slightly coarser near epistomal suture. Pubescence dense and erect in anterior regions, sparse and recumbent posteriad. Eyes slightly convex, visible from above; upper margin of eyes strongly bordered, margin anteriorly almost reaching insertion of antennae, extending posterior of eyes although weaker. Surface of head posterior to eyes flat, without depressions or fossae; surface with fine and sparse punctation, denser and coarser on clypeus; surface between punctures smooth and shiny. Antennae moniliform, strongly pubescent; pedicel oblong, brown, slightly darker than adjacent antennomeres; antennomeres longer than wide, brown, darker distally, terminal antennomeres asymmetrical and darker than pedicel; pubescence brown, of two series: first series composed of one to two pairs of long and erect pubescence per antennomere, about as long as antennomere and second series composed of denser, shorter, paler, recumbent pubescence.

***Pronotum*:** transverse, slightly paler on sides than on disc, distinctly wider at base; anterior margin of pronotum slightly concave, but almost straight between eyes, without crenulations, bordered; lateral margins only slightly arched, posterolateral angle 75–80°, with prominent borders; posterior margin with a distinct double sinuation; PL/PW = 0.42 (0.40–0.43); PW/PL = 2.40 (2.30–2.51). Punctation dense, minute and shallowly impressed; punctures larger than that of the head, denser at median line and posterior margins, sparse near suture and anteriad; surface between punctures rugose. Pubescence similar to the erect series of the head, denser on the anterior one-third of the lateral margin, evenly spaced on the rest of the margin, very sparse to almost obsolete and slightly decumbent proximally. Hypomeron flat, without depressions or fossae.

***Elytra*:**EL/EW = 1.36 (1.30–1.43); EL/PL = 3.63 (3.63–3.85); EW/PW = 1.23 (1.16–1.27); TL/EW = 1.78 (1.66–1.78), slightly less than 4.0 times longer than the pronotum, widest at anterior 0.25; anterior margin of elytra bordered, bi-sinuately articulated with pronotum; lateral margins slightly explanate in anterior half; apices jointly rounded; humeral callus weak. Elytra with two series of punctation; first series with nine or more perceptible and irregular rows of large, deeply impressed punctures; punctures of medial five rows denser and more strongly impressed in anterior half; increasingly scattered, finer and more shallowly impressed laterally and posteriorly; intervals and interstices distinctly broader than punctures; second series of punctures much smaller, densely and evenly distributed over entire elytra. Pubescence long, yellowish-brown to brown, of two distinct types (erect and recumbent): erect series on sides, slightly recumbent series on disc; erect series longer and denser; recumbent series shorter, very sparse. Scutellum subtriangular, with irregular and densely punctured surface; with two series of punctation, few large and deeply impressed punctures, numerous finer and shallowly impressed ones; pubescence erect, sparse. Metathoracic wings well developed. Epipleura almost flat.

***Ventral surface*:** punctation dense and almost uniform; pubescence brown, minute, long, finer than on dorsal side, recumbent, dense and evenly distributed. Prosternum slightly impressed at the process; process narrow, punctation more distinct at tip. Mesosternal ridge along posterior margins of mesosternum distinct. Metasternum perforate at sides, with raised triangular, rugulose area behind mesocoxal cavities; raised area comprising nearly half of surface; metasternal ridge along posterior margin of metasternum faint laterally, well-developed medially. Abdominal punctation finer at mid-line than at sides; surface between punctation with polygonal network, with median pore. Intermetacoxal plate on ventrite I triangular, strongly acuminate. Abdominal ventrite I with depressions for reception of metafemora and metatibiae; ventrites I–III connate, fused; ventrites IV–V without polygonal network; ventrite V distinctly emarginate.

***Legs*:** less than half of body length. Tibia brown, lateral margins darker, distal margin with comb of long spines; protibia very short, a little longer than half of either mesotibia and metatibia, lateral margin slightly concave, setae denser than on mesotibia and metatibia; mesotibia with lateral margin curved more prominently in interior margins, setae evenly distributed; metatibia almost twice as long as protibia, slightly longer than mesotibia, lateral margins almost parallel, setae sparse and almost recumbent; apex of mesotibia and metatibia smoothly and broadly curved. Tarsi 5-5-5, brown, paler towards the apex, almost half as long as mesotibia; tarsomere length ratio ca. 1.0:1.0:1.0:1.0:4.0 (0.9–1.1: 0.9–1.1: 0.9–1.1: 0.9–1.1: 3.5–4.7); tarsomere 1 widest towards the apex, distal margin almost double the width of proximal margin, with dense comb of setae; tarsomeres 2–4 similar to tarsomere 1, but outer edge with long yellow spiny setae on both sides, remaining portions with sparse minute setae; tarsomere 5 widest towards the apex, almost triangular, with long robust spiny setae. Tarsal claws long, narrow, symmetrical.

**Figures 4–7.**
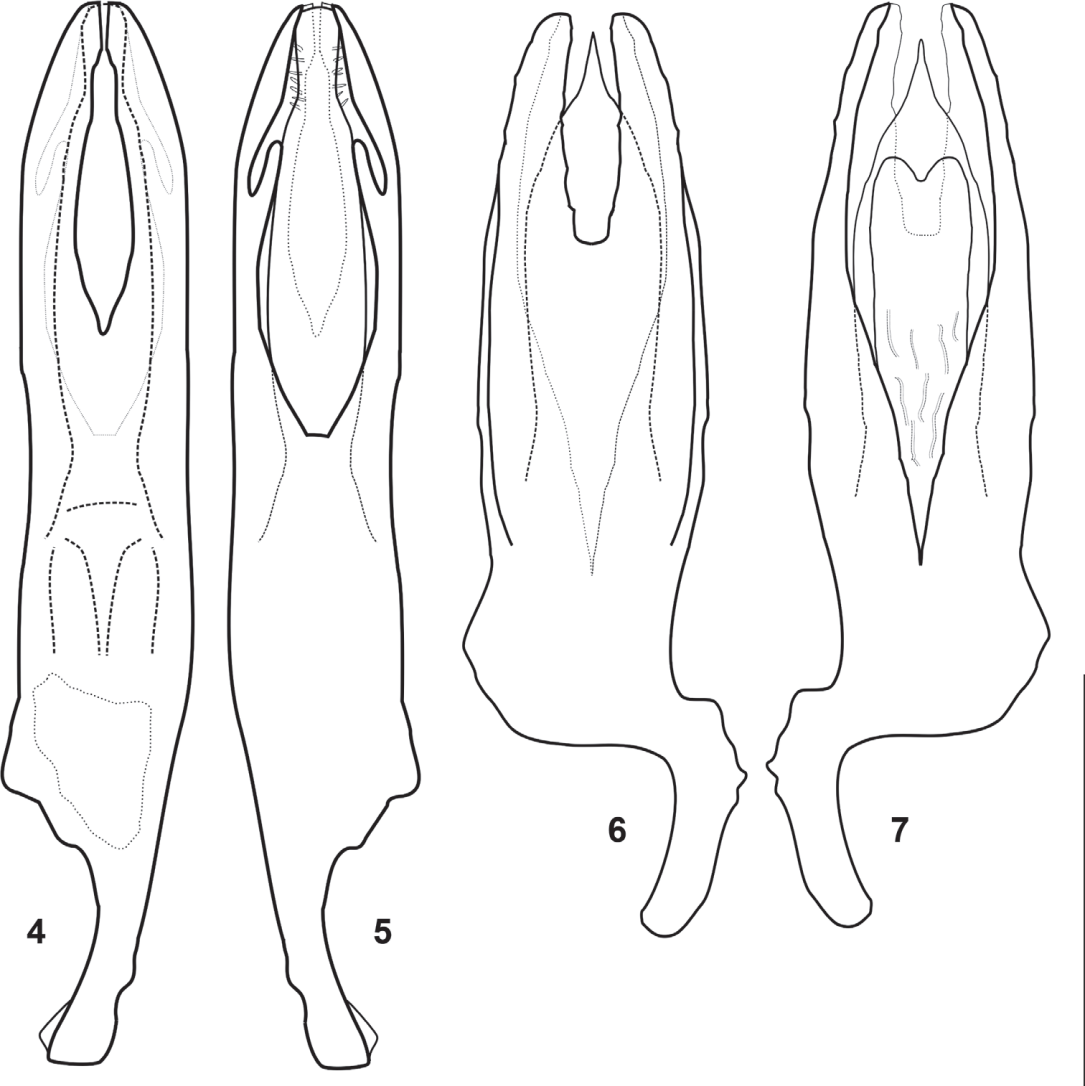
Male aedeagi of **4, 5***Byrrhinusnegrosensis* sp. nov. (EDD122) and **6, 7***Byrrhinusvillarini* sp. nov. (EDD121) in (**4, 6**) dorsal and (**5, 7**) ventral views. Scale bar: 0.25 mm.

***Male genitalia*:** (Figs 4, 5) length 0.67 mm (0.65–0.71 mm), width 0.11 mm (0.10–0.14 mm), very slender, strongly sclerotised; median lobe more exposed in ventral view than dorsal view. Median lobe of aedeagus almost as long as parameres, symmetrical; apex flat, broad, most slender subapically, with pair of rows of short denticles subapically (ventral view), convexly widened basally; basal portion wider than apical portion. Parameres symmetrical; apices slender and moderately separated dorsally, broader and converged ventrally, inner margin of parameres subparallel near tips, distinctly concave in the middle converging basally in V-shape; with tubular lobes protruding medio-apically in apical third below the denticles of the median lobe, median gap wider dorsally and exposing the full width of median lobe. Basal lobe asymmetrical, with strongly sclerotised basal margins. Ventrite VIII U–shaped, with narrow apical membranous lamina. Spiculum prominent.

***Female genitalia*:** ovipositor relatively short (0.58–0.62 mm long), straight.

####### Differential diagnosis.

In the elongate oval shape, the new species resembles several species, including *B.ferax* and *B.tarawakanus*. Amongst the Philippine species, the range of size overlaps with *B.subtestaceus* and *B.ferax*. The protibia of *B.negrosensis* sp. nov. is notably smaller than the mesotibia and metatibia. The male genitalia of *B.negrosensis* sp. nov. resembles that of *B.ferax* due to the medially parallel paramere apices, which is quite uncommon in the Oriental members of the genus. Despite numerous similarities, *B.negrosensis* sp. nov. differs from *B.ferax* in the dorsally V-shaped basal fusion point of the parameres (Fig. 4), while the latter possesses a U-shape parameral fusion. *B.negrosensis* sp. nov. varies by 13.2% mean genetic distance (723 bp *COI-3*’ mtDNA barcode) from the most similar *B.ferax* and by at least 16.1% from any other Philippine congener with available barcode (Suppl. material 1: Table S1).

####### Distribution.

This species is only recorded from the Island of Negros in the Philippines.

####### Remarks.

No external sexual dimorphism is observed. Teneral specimens are significantly paler brown.

####### Etymology.

The species is named after the Island of Negros from where the specimens were collected.

###### Byrrhinus villarini sp. nov.

Taxon classificationAnimaliaColeopteraLimnichidae

0EEF697A-4307-56AA-AC48-77A734214F7A

http://zoobank.org/45EC216F-0B75-4550-B9DF-123B9672A7FA

Figures 3 , 6 , 7

####### Type locality.

Philippines • Negros Island, Occidental Mindoro, Murcia, Pandanon River in secondary vegetation; ca. 10°34'54"N, 123°10'30"E; ca. 440 m a.s.l.

####### Type material.

***Holotype*:** Philippines • ♂ (PNM: EDD121), “PHIL: Negros Occ., Murcia, \ Pandanon River, sec.veg.; \ ca. 10°34'54"N; 123°10'30"E; ca. 440 m a.s.l.; \ 01 May 2019, leg. Freitag et al. (650)L”; GenBank: OK316813; BOLD: COLPH061-21; EDD121, habitus and terminal parts of abdomen including aedeagus glued separately on to entomological card. ***Paratypes***: Philippines • 3♂♂, 4♀♀ (AdMU: EDD113, EDD114; PNM; ZMB: EDD115): same locality data as holotype; GenBank: OK316804, OK316815, OK316817; BOLD: COLPH058-21, COLPH059-21, COLPH060-21 • 3♂♂ (AdMU: EDD124, EDD126): “PHIL: Negros Or., Valencia, \ Casaroro River, downstr., sec.veg.; \ ca. 09°18'N; 123°14'E; ca. 150 m a.s.l.; \ 01 Sep. 2019, leg. Garces & Pelingen (655)L”; GenBank: OK316805, OK316818; BOLD: COLPH062-21, COLPH063-21.

####### Description.

***Body*:** (Fig. 3) ovoid, TL = 2.8 mm (2.2–2.9 mm), EW = 1.9 mm (1.4–1.9 mm), widest behind mid-leg; dorsal surface very dark–brown to black; body appendages slightly paler than body, moderately densely and evenly covered with brown, fine, quite long, mostly erect pubescence; antennae yellow–brown; femora and tibiae brown; tarsi dark yellowish, but darker on terminal ends of segments.

***Head*:** obscurely rugulose; broadly laminate over eyes; margins of frons grooved over eyes; sides of frons with deep and well–marked pit-like depressions. Punctation minute, slightly coarser near epistomal suture. Pubescence brown, fine, quite long, erect, more numerous and denser on the anterior region. Eyes slightly convex, visible from above; upper margin of eyes bordered; anterior margin almost reaching insertion of antennae, extending posterior of eyes although weaker. Surface of head posterior to eyes flat, without depressions or fossae; surface with fine and sparse punctation, denser and coarser on clypeus; surface between punctures smooth and shiny. Antennae moniliform, strongly pubescent; pedicel globular, brown, darker than adjacent antennomeres; antennomeres longer than wide, yellow-brown; pubescence brown, uniform, erect, mostly as long as antennomere.

***Pronotum*:** transverse, black, with dark brown colouration on the sides, distinctly narrower at base; anterior margin of pronotum straight, without crenulations, bordered; lateral margins strongly arched, posterolateral angle ca. 50°, with prominent borders; posterior margin with distinct double sinuation; PL/PW = 0.42 (0.40–0.44); PW/PL = 2.40 (2.28–2.50). Punctation strong and deeply impressed, but sparse; punctures stronger than that of the head, larger at posterior margins, sparse near suture and anteriad, surface very depressed at projections along posterior margin. Pubescence similar to that on the head, slightly decumbent near the median line, denser at sides. Hypomeron flat, without depressions or fossae.

***Elytra*:**EL/EW = 1.41 (1.30–1.41); EL/PL = 4.14 (4.11–4.14); EW/PW = 1.20 (1.20–1.21); TL/EW = 1.75 (1.68–1.78); elytra slightly more than 4.0 times longer than the pronotum; widest at anterior 0.2; anterior margin of elytra bordered, strongly bi-sinuately articulated with the pronotum; lateral margins pronounced, finer towards apex; apices jointly rounded; humeral callus weak. Elytra punctation of two series; first series with nine or ten distinct and almost regular rows of large deeply impressed punctures; increasingly scattered, finer, not as strongly impressed as in rows laterally and posteriorly; intervals and interstices distinctly broader than punctures; second series of small punctures moderately dense only and less conspicuous than in previous species. Pubescence long, brown, with yellowish shade depending on illumination, of two distinct types: erect series on sides, slightly recumbent series on disc; erect series longer and denser; disc series shorter, sparse. Scutellum subtriangular, with few punctures and pubescence similar to surrounding area of elytra. Metathoracic wings well developed. Epipleura almost flat.

***Ventral surface*:** punctation dense and uniform; pubescence brown, minute, long, finer than on dorsal side, recumbent, dense and evenly distributed. Prosternum slightly impressed at the process; process narrow, punctation more distinct at tip. Mesosternal ridge along posterior margins distinct. Metasternum minutely perforate at sides, with raised triangular, rugulose area behind cavities; raised area comprising nearly half of surface; metasternal ridge along posterior margin of metasternum faint laterally, well-developed medially. Abdominal punctation finer at mid-line than at sides; surface between punctation with polygonal network, with median pore. Intermetacoxal plate on ventrite I triangular, strongly acuminate. Abdominal ventrite I with depressions for reception of metafemora and metatibiae; ventrites I–III connate, fused; ventrites IV–V without polygonal network; ventrite V distinctly emarginate apically.

***Legs*:** length less than half of body length. Tibia brown, lateral margins darker, curved, with pre-apical comb of spines; metatibia slightly longer than protibia and mesotibia; apex of mesotibia and metatibia slightly curved. Tarsi 5-5-5, dark yellow to light brown, paler towards the apex, about two-thirds of length of tibia; tarsomere length ratio ca. 1.2:1.0:1.0:1.0:2.9 (1.0–1.5: 0.8–1.0: 0.8–1.0: 0.8–1.0: 2.7–3.3); tarsomere 1 brown, with parallel margins, widest towards the apex, with dense comb of setae; tarsomeres 2–4 almost globular, outer edge with long yellow spiny setae on both sides, remaining portions with sparse minute setae; tarsomere 5 more yellow than brown, widest towards the apex, with at least three pairs of long robust spiny setae. Tarsal claws long, narrow, symmetrical.

***Male genitalia*:** (Figs 6, 7) length 0.58 mm (0.57–0.59 mm), width 0.14 mm (0.12–0.17 mm), stout, strongly sclerotised; median lobe more exposed in ventral view than dorsal view. Median lobe of aedeagus a bit shorter than parameres, symmetrical, broad, non-planar, varying in dorsal and ventral views; on dorsal view, apex acuminate, significantly and abruptly convexly widened mediad, middle portion wide; on ventral view, median lobe with an additional, slightly more slender, subcordiform lobe, reaching apical 0.3 where it terminates deeply emarginate. Parameres symmetrical, with outer face irregularly outlined and uneven texture near apex; apices dorsally broad; apices obliquely rounded; inner margins dorsally very slightly concave, almost unevenly sub-parallel; not distinctly convergent basally, forming a U–shape extending one-fourth the length of genitalia; ventrally only half as wide as in dorsal view, opening wider and exposing the entire width of median lobe, converging basally to form a deep “V”. Basal lobe asymmetrical, with strongly sclerotised basal margins. Ventrite VIII U–shaped, with narrow apical membranous lamina. Spiculum prominent.

***Female genitalia*:** ovipositor relatively short (0.50–0.56 mm long), straight.

####### Differential diagnosis.

In the ovoid shape, the new species resembles *B.vestitus* (Sharp, 1902), *B.maculatus* Wooldridge, 1987 and *B.magnus* Wooldridge, 1987. Compared to other Philippine species, the range of size overlaps with *B.punctatus* and *B.tarawakanus*. The new species is remarkably different from these two in the smaller posterolateral angle (ca. 50°) of the pronotum in *B.villarini* sp. nov. compared to *B.punctatus* and *B.tarawakanus*, as well as *B.negrosensis* sp. nov. (75–80°). In addition to the posterolateral angle measure of pronotum, *B.villarini* sp. nov. is notably different from *B.negrosensis* sp. nov. in the length of tarsomere 5. Tarsomere 5 of *B.villarini* sp. nov. is as long as tarsomeres 2–4, while tarsomere 5 of *B.negrosensis* sp. nov. is almost as long as tarsomeres 1–4. Additionally, erect series of elytral pubescence is present on the posterior end of both species, but covers the distal one-third only of elytra in *B.villarini* sp. nov., while covering the distal one-half in *B.negrosensis* sp. nov.

Males of *B.villarini* sp. nov. are easily recognisable because the parameres are dorsally fused forming a rather shallow “U” (Fig. 6), not a “V” as in *B.negrosensis* sp. nov. (Fig. 4). This U–shaped opening separating the parameres extends only one-fourth the length of the aedeagus, while the opening spans at least half of the aedeagus for other Philippine species, such as *B.ferax*, *B.punctatus* and *B.tarawakanus*. The median lobe of the aedeagus resembles that of *B.tarawakanus* in terms of shape and height of the parameres. However, the median lobe of *B.villarini* sp. nov. is stouter and wider towards the middle portion.

*B.villarini* sp. nov. varies by 5.8% mean genetic distance (723 bp *COI-3*’ mtDNA barcode) from an unidentified, but presumably closely-related Malaysian species and by at least 13.4% from any other Philippine congeners with available barcodes (Suppl. material 1: Table S1).

####### Distribution.

This species is only recorded from the Island of Negros in the Philippines.

####### Remarks.

No external sexual dimorphism is observed.

####### Etymology.

The new species is named and dedicated to the immediate past president of the Ateneo de Manila University, Fr Jose Ramon T. Villarin, SJ, PhD, who finished his term last year. During his reign for the past decade, Fr Villarin showed ardent support for research activities on the environment and sustainability. He is also a member of the Intergovernmental Panel on Climate Change which was conferred the 2007 Nobel Peace Prize for their study and recommendations on counteracting the global climate crisis.

###### Byrrhinus ferax

Taxon classificationAnimaliaColeopteraLimnichidae

Wooldridge, 1993

C4A7944C-2EDE-5828-A8AF-9862F6FEC9CF

Byrrhinus ferax Wooldridge, 1993: 359–360 (orig. descr.).

####### Additional material examined.

Philippines • ♂ (AdMU), Occ. Mindoro, Sablayan, small limestone river; rootpacks, dist. primary forest; ca. 12°47'49"N, 120°54'33"E; ca. 100 m a.s.l.; 01 Jan. 1995, leg. Mendoza “(365)M”; GenBank: OK316810; BOLD: COLPH064-21.

####### Remarks.

Assignment of the specimen to this taxon was based primarily on genital characters. No remarkable difference was noted compared to the original description. The material used for the molecular analysis in this study was collected 90 km south of one of the localities of the paratypes, but on the same island. Known distribution of *B.ferax* includes the Philippine Islands of Mindoro and Mindanao.

#### Additional *Byrrhinus* specimens examined

The three specimens listed below have an interspecific divergence of 10.6 to 19.6% (Table 2). Given the single-specimen samples that are currently available only, they will not receive further treatment in this study.

***Byrrhinus* sp. A**: Philippines • 1 ♂, Palawan, P. Princesa, Irawan River, 6 km NW of PPC, 0.5 km upstream of water plant ca. 9°49'50"N, 118°39'46"E; 105 m a.s.l.; 06 Aug. 2019, leg. H. Freitag “(60b)M”; GenBank: OK316816; BOLD: COLPH065-21.

***Byrrhinus* sp. B**: Philippines • 1 ♂, Camarines Sur, Lupi, Brgy. Sooc, Sooc River, Bicol National Park; ca. 13°52'28"N, 122°56'38"E; 90 m a.s.l.; 09 Aug. 1996; leg. Mendoza “(M585)L”; GenBank: OK316806; BOLD: COLPH066-21.

***Byrrhinus* sp. C**: Philippines • 1 ♂, Mindanao, Agusan N, R.T.R, Panaytayon, paddy field, ca.10 m a.s.l. 9°02'53"N, 125°35'03"E; leg. Freitag & Pangantihon 05. Jul. 2018 “(890)L”; GenBank: OK316814; BOLD: COLPH067-21.

### Checklist and distribution of the species of *Byrrhinus* in the Philippines

*Byrrhinusconvexus* (Blackburn, 1896): Luzon, Australia

*Byrrhinusferax* Wooldridge, 1993: Mindoro, Mindanao (Cotabato, Davao); Borneo (Malaysia: Sabah)

*Byrrhinusnegrosensis* sp. nov.: Negros

*Byrrhinuspunctatus* (Pic, 1922): Luzon, Mindoro

*Byrrhinusvillarini* sp. nov.: Negros

*Byrrhinussubtestaceus* Pic, 1923: Luzon

*Byrrhinustarawakanus* Deléve, 1973: Palawan, Tawi-Tawi

## Discussion

**Figure 8.**
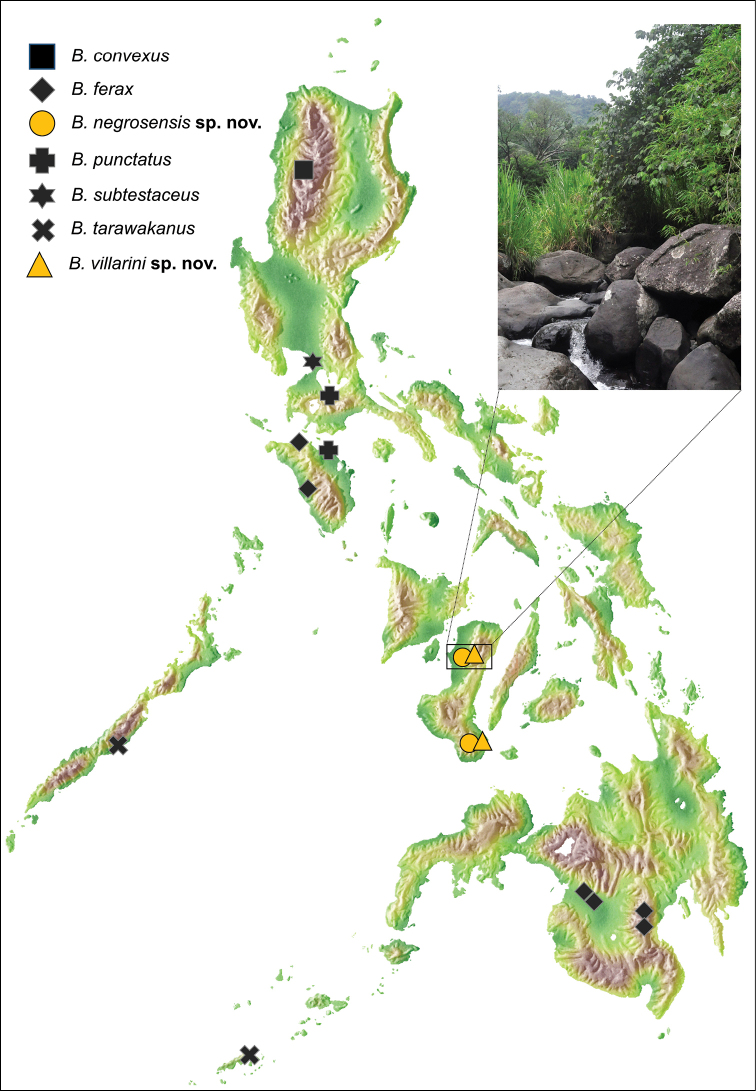
Updated distribution of known *Byrrhinus* in the Philippines; inset shows Pandanon River in Murcia “(650)L”, which is the type locality of *B.villarini* sp. nov.

The two new *Byrrhinus* species described here, *B.negrosensis* sp. nov. and *B.villarini* sp. nov., increase the total number of Philippine *Byrrhinus* from five to seven. This contribution using the integrative taxonomic approach provides the first additional record on Philippine *Byrrhinus* species in the last 28 years and provides the first records from the Visayas (Fig. 8; see updated checklist). This study demonstrates that sampling in one of the several islands of Visayas reveals previously undocumented entomofauna.

Characters used to differentiate limnichid species, such as pubescence and punctation patterns and aedeagal structure (Hernando and Ribera 2014a, 2014b), were notably different for the two Negros *Byrrhinus* species as described above. From the materials studied, *B.villarini* sp. nov. is smaller (2.1–2.9 mm) than *B.negrosensis* sp. nov. (2.9–4.1 mm) though these size ranges are not unique compared other *Byrrhinus* species. While the usual body ratios (PL/PW, EL/EW, EL/PL, EW/PW, TL/EW) applied to Limnichidae (Yoshitomi 2019) did not substantially help discriminating the two new species, the length ratios of the tarsomeres and the posterolateral angle of the pronotum appear to be distinct. Female specimens show no distinct differences from the male external morphology.

Phylogenetic analysis retrieved strongly supported clades (BS = 100) corresponding to the two newly-described species (Fig. 1). Each species was poorly clustered (BS < 70) with *Byrrhinus* from other islands. The sequences of *B.negrosensis* sp. nov. clustered with the first *COI-3*’ sequence for *B.ferax*. Moreover, the sequences for *B.villarini* sp. nov. clustered with *Byrrhinus* sp. from Malaysia in a well-supported clade. While the genetic distance is low at 5.9% (Table 2), it is beyond the recognised 3% rule-of-thumb for intraspecific divergence limit for insects (Hebert et al. 2003; Astrin et al. 2012). The said sequence, together with the other reference sequences used in this analysis, was generated in a study on resolving superfamily phylogeny (Kundrata et al. 2017). As the sequence was not accompanied by a proper species-level identification or informative photographs on BOLD, no further discussion can be provided on this clade of presumably closely-related species.

**Figure 9.**
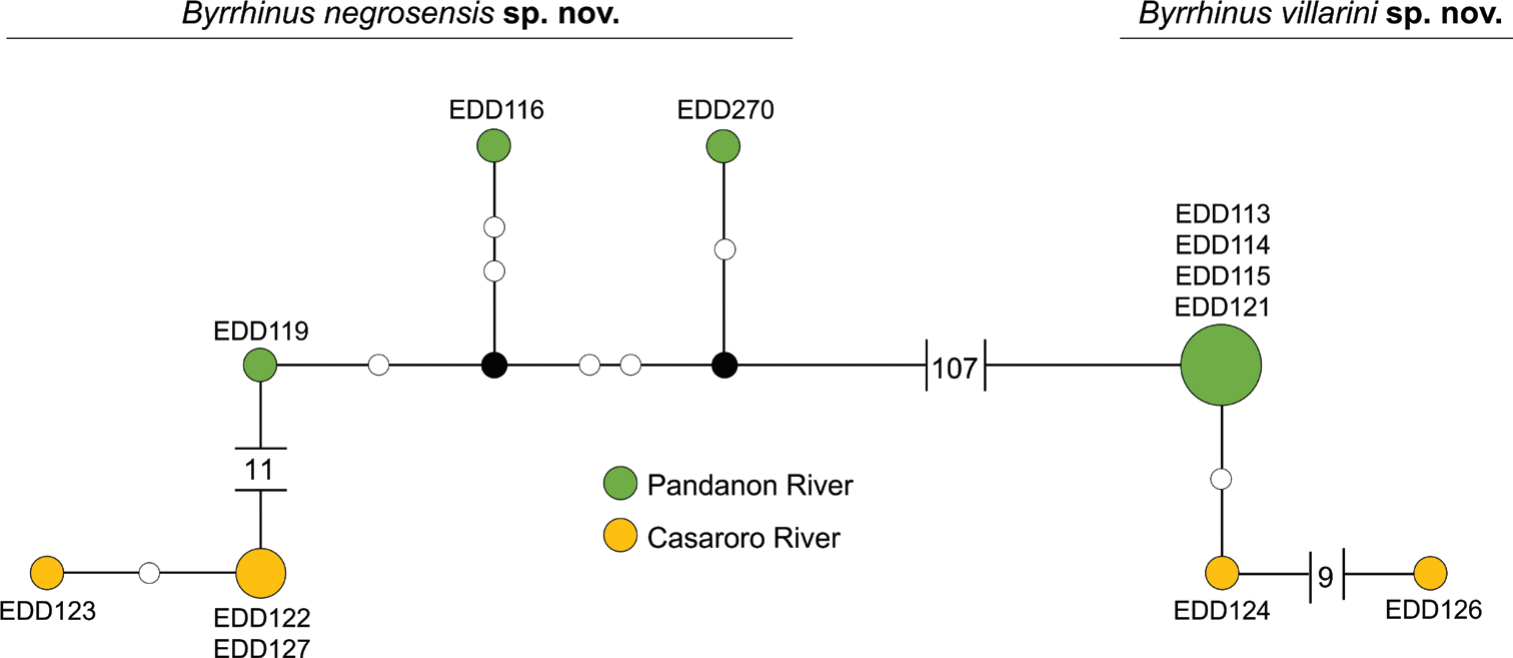
Statistical parsimony network of sequenced *B.negrosensis* sp. nov. and *B.villarini* sp. nov. specimens using *COI-3*’ (723 bp).

The clustering of conspecific sequences in the gene tree is also reflected in the statistical parsimony network generated for the two new species (Fig. 9). Conspecific sequences from different localities are less divergent than sequences of syntopic, but non-conspecific specimens. The gene tree (Fig. 1) also shows that the two new Negros species are genetically closer to some other *Byrrhinus* species which are not (yet) recorded from Negros than with each other.

Entomofaunal diversity has been experiencing a tremendous decline in both local (Hallmann et al. 2021; Warren et al. 2021) and global scales (Sánchez-Bayo and Wyckhuys 2019), with catastrophic impacts on the current ecosystem services (Faridah-Hanum et al. 2018; Wagner et al. 2021). Especially in time of accelerated biodiversity decline, coupling morphological and molecular data is essential in fast-tracking species discovery particularly in highly cryptic and highly diverse taxa (Tänzler et al. 2014; Platania et al. 2020; Maasri et al. 2021). In such cases, proper taxonomic identification of cryptic taxa using an integrative approach has been shown to be essential in mapping appropriate conservation measures (Arribas et al. 2013). These discoveries came at a time of increased anthropogenic activities in the protected areas and highly pristine localities in the Island.

## Supplementary Material

XML Treatment for Byrrhinus negrosensis

XML Treatment for Byrrhinus villarini

XML Treatment for Byrrhinus ferax

## Supplementary materials

Supplementary material 1 Data type Genetic distance
**Table S1**
Explanation note **Table S1.** Kimura 2-parameter (K2P) genetic distance of the samples of *Byrrhinus* specimens, based on their aligned partial *COI-3*’ sequences of 723 bp length (in %). https://binary.pensoft.net/file/609408This dataset is made available under the Open Database License (http://opendatacommons.org/licenses/odbl/1.0/). The Open Database License (ODbL) is a license agreement intended to allow users to freely share, modify, and use this Dataset while maintaining this same freedom for others, provided that the original source and author(s) are credited.Emmanuel D. Delocado, Hendrik Freitag
