# Supplementary material for: Two new species of Byrrhinus Motschulsky, 1858 (Coleoptera, Limnichidae, Limnichinae) from Negros, Philippines
Source: Zookeys. 2021 Nov 10;1070:51–72. doi: 10.3897/zookeys.1070.70531 (PMC8599385; doi:10.3897/zookeys.1070.70531)
Supplement: Supplementary material 1 [file zookeys-1070-051-s001.docx]

**Supplementary File 1.**

**Table S1.** Kimura 2-parameter (K2P) genetic distance of the samples of *Byrrhinus* specimens based on their aligned partial *COI-3’* sequences of 723 bp length (in %).

|  |  | EDD116 | EDD119 | EDD122 | EDD123 | EDD127 | EDD270 | EDD113 | EDD114 | EDD115 | EDD121 | EDD124 | EDD126 | EDD067 | EDD057 | EDD105 | EDD112 | UPOL RK0727 | UPOL RK0664 |
| --- | --- | --- | --- | --- | --- | --- | --- | --- | --- | --- | --- | --- | --- | --- | --- | --- | --- | --- | --- |
| EDD116 | *Byrrhinus negrosensis* sp. nov. |  |  |  |  |  |  |  |  |  |  |  |  |  |  |  |  |  |  |
| EDD119 | *Byrrhinus negrosensis* sp. nov. | 0.4 |  |  |  |  |  |  |  |  |  |  |  |  |  |  |  |  |  |
| EDD122 | *Byrrhinus negrosensis* sp. nov. | 2.0 | 1.6 |  |  |  |  |  |  |  |  |  |  |  |  |  |  |  |  |
| EDD123 | *Byrrhinus negrosensis* sp. nov. | 2.2 | 1.7 | 0.1 |  |  |  |  |  |  |  |  |  |  |  |  |  |  |  |
| EDD127 | *Byrrhinus negrosensis* sp. nov. | 2.0 | 1.6 | ― | 0.1 |  |  |  |  |  |  |  |  |  |  |  |  |  |  |
| EDD270 | *Byrrhinus negrosensis* sp. nov. | 0.7 | 0.6 | 2.2 | 2.3 | 2.2 |  |  |  |  |  |  |  |  |  |  |  |  |  |
| EDD113 | *Byrrhinus villarini* sp. nov. | 19.5 | 19.5 | 19.5 | 19.7 | 19.5 | 19.0 |  |  |  |  |  |  |  |  |  |  |  |  |
| EDD114 | *Byrrhinus villarini* sp. nov. | 19.5 | 19.5 | 19.5 | 19.7 | 19.5 | 19.0 | ― |  |  |  |  |  |  |  |  |  |  |  |
| EDD115 | *Byrrhinus villarini* sp. nov. | 19.5 | 19.5 | 19.5 | 19.7 | 19.5 | 19.0 | ― | ― |  |  |  |  |  |  |  |  |  |  |
| EDD121 | *Byrrhinus villarini* sp. nov. | 19.5 | 19.5 | 19.5 | 19.7 | 19.5 | 19.0 | ― | ― | ― |  |  |  |  |  |  |  |  |  |
| EDD124 | *Byrrhinus villarini* sp. nov. | 19.7 | 19.7 | 19.7 | 20.0 | 19.7 | 19.2 | 0.1 | 0.1 | 0.1 | 0.1 |  |  |  |  |  |  |  |  |
| EDD126 | *Byrrhinus villarini* sp. nov. | 20.7 | 20.7 | 20.7 | 21.0 | 20.7 | 20.0 | 1.3 | 1.3 | 1.3 | 1.3 | 1.1 |  |  |  |  |  |  |  |
| EDD067 | *Byrrhinus ferax* Wooldridge, 1993 | 13.4 | 13.6 | 13.0 | 12.8 | 13.0 | 13.2 | 13.8 | 13.8 | 13.8 | 13.8 | 14.0 | 13.4 |  |  |  |  |  |  |
| EDD057 | *Byrrhinus* sp. A | 19.7 | 19.9 | 19.7 | 20.0 | 19.7 | 19.5 | 16.0 | 16.0 | 16.0 | 16.0 | 16.2 | 17.1 | 19.5 |  |  |  |  |  |
| EDD105 | *Byrrhinus* sp. B | 16.3 | 16.1 | 16.8 | 17.0 | 16.8 | 16.8 | 15.6 | 15.6 | 15.6 | 15.6 | 15.4 | 15.6 | 19.4 | 17.7 |  |  |  |  |
| EDD112 | *Byrrhinus* sp. C | 18.2 | 18.2 | 18.0 | 18.2 | 18.0 | 18.4 | 17.2 | 17.2 | 17.2 | 17.2 | 17.4 | 18.6 | 21.4 | 10.6 | 19.6 |  |  |  |
| UPOL RK0727 | *Byrrhinus* sp. (Malaysia) | 20.2 | 20.2 | 19.7 | 20.0 | 19.7 | 20.2 | 5.8 | 5.8 | 5.8 | 5.8 | 6.0 | 6.1 | 17.0 | 16.4 | 17.0 | 13.7 |  |  |
| UPOL RK0664 | *Byrrhinus* sp. (Indonesia) | 21.8 | 22.1 | 21.1 | 21.4 | 21.1 | 22.1 | 20.0 | 20.0 | 20.0 | 20.0 | 20.3 | 19.5 | 21.7 | 22.2 | 22.4 | 17.9 | 20.2 |  |
| UPOL RK0663 | *Byrrhinus* sp. (Cameroon) | 25.1 | 25.1 | 25.1 | 25.4 | 25.1 | 25.4 | 28.7 | 28.7 | 28.7 | 28.7 | 29.0 | 29.0 | 28.6 | 24.7 | 25.0 | 26.5 | 28.9 | 23.4 |
